# Advanced Finite Element Analysis Process for Accurate Cured Tire Shape Forecasting

**DOI:** 10.3390/polym17111546

**Published:** 2025-06-01

**Authors:** Sairom Yoo, Hyunseung Kim, Yongsu Kim, Kideug Sung, Hyeonu Heo

**Affiliations:** 1 Virtual Research Department, Design Analysis Team, Nexen Tire Corp, 177, Magokjungang-ro, Gangseo-Gu, Seoul 157-010, Republic of Korea; 10221008@nexentire.com (H.K.); yskim@nexentire.com (Y.K.); kdsung36@nexentire.com (K.S.); 2 Department of Mechanical Engineering, The University of Akron, 244 Sumner Street, Akron, OH 44325-3903, USA

**Keywords:** tire, post-cure inflation, fabric cord, thermal shrinkage, permanent set, finite element analysis

## Abstract

Tire shape prediction presents significant engineering challenges due to the complex behavior of cord-rubber composites during manufacturing processes. Fabric cord components undergo thermal shrinkage and permanent deformation that substantially influence final tire dimensions, creating discrepancies between mold geometry and cured tire shape. While Post-Cure Inflation (PCI) helps control these dimensional changes, accurate prediction methods remain essential for reliable performance forecasting. This study addresses this challenge through a systematic experimental characterization of fabric cord behavior under manufacturing conditions. Thermal shrinkage and permanent set were quantified under various combinations of in-mold strain and PCI force, with distinct patterns identified for different cord materials (PET and nylon). Based on these experimental findings, a comprehensive finite element analysis methodology was developed to predict cured tire shape. Validation against 65 tire profiles demonstrated remarkable improvements over conventional approaches, with dimensional error reductions of 54.2% for the outer diameter and 49.5% for the section width. Profile and footprint predictions also showed significantly enhanced accuracy, particularly in capturing geometric features critical for tire–road contact characteristics. The proposed methodology enables more precise tire design optimization, improved performance prediction, and reduced prototype iterations, ultimately enhancing both product development efficiency and final tire performance.

## 1. Introduction

Tires are complex composite material products composed of fabric cord, steel cord, and rubber compound. These heterogeneous materials are integrated, and the rubber is hardened through a vulcanization process [1]. The vulcanization process takes place at high temperatures during the curing stage, which occurs inside the mold. Although the mold determines the tire’s overall shape, various factors during the process can cause the final tire shape to deviate from the intended mold geometry.

The final shape of the tire, commonly referred to as the cured tire shape, typically differs from the mold shape due to several key factors [2]:

➀ Thermal shrinkage of rubber compounds.
➁ Thermal shrinkage and expansion of fabric cord.
➂ Permanent deformation of fabric cord.
➃ Modulus change in fabric cord due to high-temperature exposure.

The thermal shrinkage and permanent deformation of the fabric cord, which constitutes the tire’s primary structural framework (body ply), have been identified as the most significant contributors to cured tire shape deviation [3,4]. These dimensional changes not only affect the tire’s geometry but also significantly impact its performance characteristics, including handling, wear patterns, and rolling resistance.

Despite the critical importance of accurately predicting cured tire shape, research on the effects of combined mechanical loading and temperature variations on fabric cord behavior remains relatively limited. Previous studies have approached this challenge from various perspectives. A tire manufacturing company investigated the deformation behavior of fabric cord in the Post-Cure Inflation (PCI), analyzing load–deformation characteristics of the cord under curing and PCI conditions to predict the deformation of the tire crown, the part of the tire that makes contact with the road [5,6]. However, this study lacked a finite element analysis (FEA) and provided insufficient information for comprehensive tire shape prediction.

Another notable study was conducted by Kennedy [2], who developed a finite element model to predict the cured tire shape by analyzing fabric cord creep behavior during PCI. While pioneering, this approach only considered loading conditions in the PCI state and was validated solely on tires with relatively high-aspect ratios (Series 65, 70, and 75). However, current trends in high-performance tire development are increasingly focused on lower aspect ratio profiles (e.g., 40, 45, and 50), which present unique challenges in shape prediction due to their geometry and higher cord tensions.

Recent advancements include Zreid et al. [7], who developed a finite element model to simulate thermal shrinkage in polymeric tire cords and the resulting dimensional changes during the PCI process. Their work demonstrated a significant impact on final tire dimensions when comparing predictions made with and without accounting for the PCI process. However, this study did not address the permanent deformation that occurs during PCI, particularly its varying effects across different tire geometries.

Korunovic et al. [8] evaluated various material models for tire cords, comparing their accuracy, computational efficiency, and parameter identification requirements. Their research highlighted the importance of selecting appropriate material models (such as linear, Yeoh, and Marlow models) for the accurate finite element analysis of tires. Importantly, they emphasized the necessity of accounting for high-temperature effects during the curing process and acknowledged that changes in cord properties throughout curing represented critical factors for accurate tire simulation.

Conventional tire shape prediction models have been developed primarily for traditional high-aspect-ratio tires and exhibit significant limitations when applied to low-aspect-ratio designs. The existing literature has not adequately addressed the fundamental differences in deformation mechanics between these tire types, creating notable research gaps. In low-aspect-ratio tires, the reduced sidewall height results in less sidewall deformation under air pressure, causing a greater proportion of deformation energy to concentrate in the tread region. This leads to more significant tread inflation compared to high-aspect-ratio tires, particularly during the PCI process. Furthermore, the process of contraction and expansion due to heating and cooling of the cord material has a more pronounced effect on low-aspect-ratio tires, yet conventional models fail to account for this increased sensitivity to thermal cycling and its effect on permanent set in reinforcement materials. Additionally, existing models inadequately capture how the mechanical properties of reinforcement materials evolve through the manufacturing process from green tire to final cured state, especially in the unique geometric configuration of low-aspect-ratio tires.

To address these limitations, this study proposes a comprehensive approach that characterizes the behavior of fabric cord creep in terms of permanent set and thermal shrinkage under the conditions that simulate the entire manufacturing process. We develop an enhanced FEA methodology using ABAQUS to more accurately predict cured tire shape across various tire geometries. The proposed model is rigorously validated against experimental measurements and demonstrates improved prediction accuracy for modern low-aspect-ratio tire designs.

## 2. Methods

### 2.1. Multi-Stage FEA Simulation of Tire Manufacturing Process

**Figure 1:**
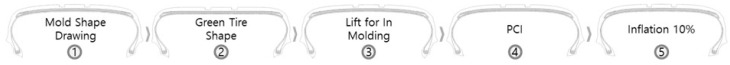
Flowchart of the FEA for predicting cured tire shape.

Previous studies have primarily focused on predicting tire shape by analyzing mechanical loads and thermal behavior during the PCI process alone. In contrast, this study introduces a more comprehensive approach. We propose that strain generated during the mold lift process, which allows the removal of the newly cured tire and occurs before PCI, significantly affects the deformation behavior of fabric cord during subsequent PCI. Figure 1 illustrates the flowchart of our proposed multi-stage FEA methodology for cured tire shape prediction.

The process began with simulating the green (uncured) tire shape for the mold lift analysis to quantitatively derive the strain occurring in the fabric cord during this critical phase, yielding in-mold strain values. Subsequently, an FEA simulation of the PCI process calculated the PCI force acting on the fabric cord, while also integrating thermal shrinkage effects to accurately model the tire shape during PCI.

To complete the methodology, we conducted experimental tests to establish the relationship between permanent set ($\varepsilon_{p}$) as a function of in-mold strain ($\varepsilon_{In-mold}$) and PCI force ($f_{pci}$). The derived permanent set values were then applied in a final simulation with an additional 10% increase in inflation pressure, allowing for the accurate prediction of the final cured tire shape. This multi-stage approach captured the cumulative effects of sequential manufacturing processes on cord deformation, providing a more realistic shape prediction than conventional methods.

The finite element model was implemented in ABAQUS 2023 with carefully selected material definitions for both rubber compounds and cord materials. For the rubber components, we incorporated a combination of hyperelastic and viscoelastic material properties. Specifically, we employed the neo-Hookean model to represent hyperelastic behavior, while the Prony series captured the time-dependent viscoelastic response. For the cord material, we implemented a multi-stage modeling approach that evolved throughout the simulation process:

- In-molding and PCI analysis: during these initial stages, the Marlow model with a 5% compression ratio was applied to accurately capture the cord’s mechanical response under manufacturing conditions.
- Inflation stage: for this subsequent phase, we transitioned to using the Plastic keyword to account for the permanent set behavior of the cord material, reflecting the irreversible deformation that occurs during manufacturing.

**Figure 2:**
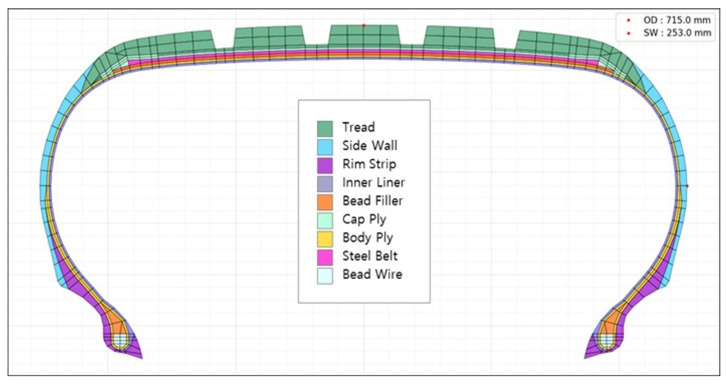
Tire structure and FEA model.

The boundary conditions were carefully defined to represent the actual tire operation. The bead wire (shown in Figure 2) was fully constrained to simulate its connection to the wheel rim. To replicate the PCI process, we applied inflation pressure to the inner surface of the tire using the DLOAD keyword in ABAQUS.

A key aspect of our approach was the systematic updating of cord material properties at each simulation stage (in-molding, PCI, and 10% inflation) to reflect the evolving mechanical behavior throughout the manufacturing process. This sequential approach allowed us to account for the progressive changes in material response due to processing conditions.

It is important to note that we did not conduct an explicit thermal analysis in this study. This decision was based on experimental observations that the temperature distribution within the tire during the curing process was relatively uniform and did not create significant locational variations that would substantially affect the cord material properties. The primary effect of temperature in tire manufacturing is on the degree of rubber curing rather than on spatial variations in cord properties.

Instead of using a full thermal–mechanical coupled analysis, we modeled thermal contraction effects using initial stress definitions in ABAQUS. This approach allowed us to effectively account for thermal shrinkage while the cord was simultaneously experiencing tensile loading during the PCI process, providing computational efficiency without sacrificing accuracy in capturing the critical thermal–mechanical interactions.

Figure 2 illustrates the key structural components of the tire. Each component serves a specific function in the overall tire performance:

- Tread (green): the outermost layer contacting the road surface, providing traction, wear resistance, and grip.
- Side wall (light blue): the lateral portion that absorbs impact forces and enhances flexibility, contributing to ride comfort.
- Rim strip (purple): creates an airtight seal between the wheel rim and tire to prevent air leakage.
- Inner liner (brown): an air-impermeable layer that maintains internal air pressure and prevents air diffusion.
- Bead filler (orange): increases bead area stiffness, enhancing steering response and handling characteristics.
- Cap ply (light green): Suppresses radial expansion due to centrifugal forces during high-speed operation. Typically constructed from nylon-based cord–rubber composite.
- Body ply (yellow): Forms the primary structural framework of the tire, supporting loads and absorbing impact forces. Commonly fabricated from PET-based cord–rubber composite.
- Steel belt (pink): a steel cord–rubber composite layer that reinforces the tread area, increasing rigidity and durability while optimizing ground contact.
- Bead wire (light sky blue): metallic wire bundle that secures the tire to the wheel rim and maintains the tire’s dimensional stability.

### 2.2. Experimental Setup for Fabric Cord

Fabric cord serves as a critical reinforcement material in tires, undergoing significant mechanical property changes when subjected to various loading conditions throughout the manufacturing process [3]. While perfectly simulating these complex transformations presents considerable challenges, a targeted experimental approach focused on key transformation phases, specifically the curing (in-molding) process and the PCI state, enables a more accurate characterization of fabric cord property variations.

**Figure 3:**
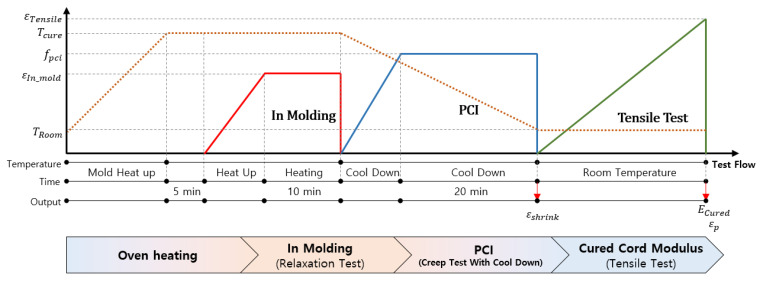
Flowchart of the fabric cord experiment, showing the evolution of temperature (*T*), in-mold strain ($\varepsilon_{e}$), and PCI force ($f_{\mathrm{PCI}}$) over time through the different stages: oven heating, in-molding relaxation, PCI creep with cool down, and final tensile testing to measure cured cord modulus ($E_{\mathrm{cured}}$) and permanent set ($\varepsilon_{p}$). The orange dotted line indicates temperature changes throughout the manufacturing process. The red, blue, and green solid lines represent the changes in in-mold strain, PCI force, and tensile test strain, respectively.

In this study, we designed an experimental apparatus to precisely replicate the property changes in fabric cord during tire manufacturing, as illustrated in Figure 3. This setup was configured to simulate both the curing (in-molding) stage and the PCI process to accurately characterize the material behavior.

The primary objective of these experiments was to determine three key material properties that significantly influence the final tire shape: permanent set ($\varepsilon_{p}$), thermal shrinkage ($\varepsilon_{sh}$), and cured modulus ($E_{cured}$).

The experimental procedure followed these steps:

➀ Mount the fabric cord specimen onto a jig and set the initial cord length ⓐ $L_{0}$.
➁ Heat up the chamber to the curing temperature to simulate the thermal effects of the curing process.
➂ Once the fabric cord reaches the target temperature, apply deformation corresponding to the in-mold strain condition.
➃ Measure the cord length ⓑ after applying in-mold strain.
➄ Remove the applied load and initiate the cooling process by turning off the chamber.
➅ During the cooling phase, apply tensile loading according to the PCI force condition.
➆ Remove the PCI force and measure the cord length ⓒ.
➇ After curing, measure the cured modulus $E_{cured}$ of the fabric cord.

**Figure 4:**
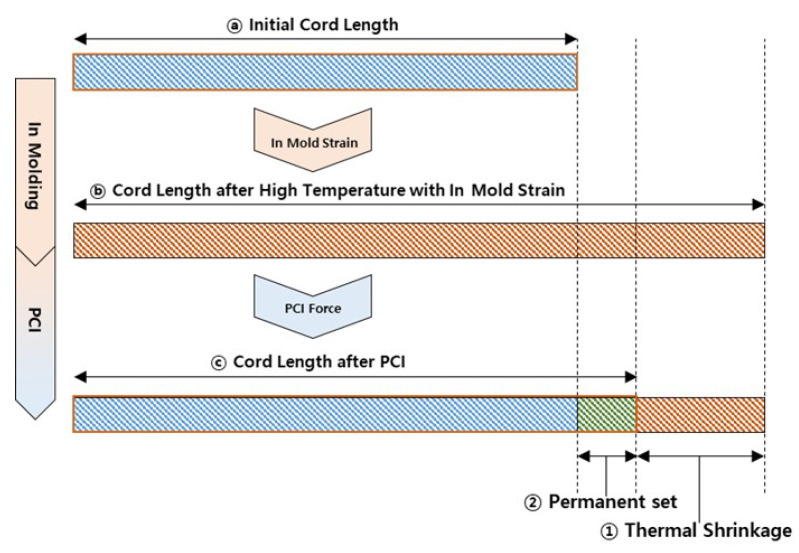
Schematic illustration of cord length changes during in molding and PCI processes. The diagram shows the initial cord length ⓐ, cord length after high temperature exposure with in-mold strain ⓑ, and cord length after PCI ⓒ. The thermal shrinkage ➀ and permanent set ➁ are clearly indicated. Blue bars represent the code length at room temperature before and after curing, and red bars represent the cord length at high temperature during curing and the green bar represent the permanent set cord length after PCI.

During the experimental process, we measured the length variations in the fabric cord to define thermal shrinkage and permanent set, as schematically illustrated in Figure 4. The thermal shrinkage ($\varepsilon_{sh}$) and permanent set ($\varepsilon_{p}$) were formulated mathematically according to the following measurements.

(1) $$\varepsilon_{sh}=\left(CordLength\;ⓑ-CordLength\;ⓒ\right)/CordLength\;ⓐ$$

(2) $$\varepsilon_{p}=\left(CordLength\;ⓒ-CordLength\;ⓐ\right)/CordLength\;ⓐ$$

We employed a universal tensile testing machine to conduct the experiment. A single cord was mounted on a cord-specific jig, and the test was carried out following the procedure outlined below. The specimen had an initial gauge length of 300 mm, and the cord length was measured at each stage as it changed, as illustrated in Figure 4.

This study investigated fabric cord behavior under varying manufacturing conditions through a test matrix with three distinct levels of in-mold strain and three levels of PCI force, as presented in Table 1. We selected these parameters to represent the range of conditions encountered during actual tire manufacturing processes.

A comprehensive set of nine experimental conditions (3 × 3 factorial design) was executed to characterize material response across the operational parameter space. Three specimens were tested under each condition to ensure statistical reproducibility. The thermal shrinkage and permanent set of the fabric cord were quantified as functions of the processing parameters, providing essential data for the subsequent finite element modeling.

**Figure 5:**
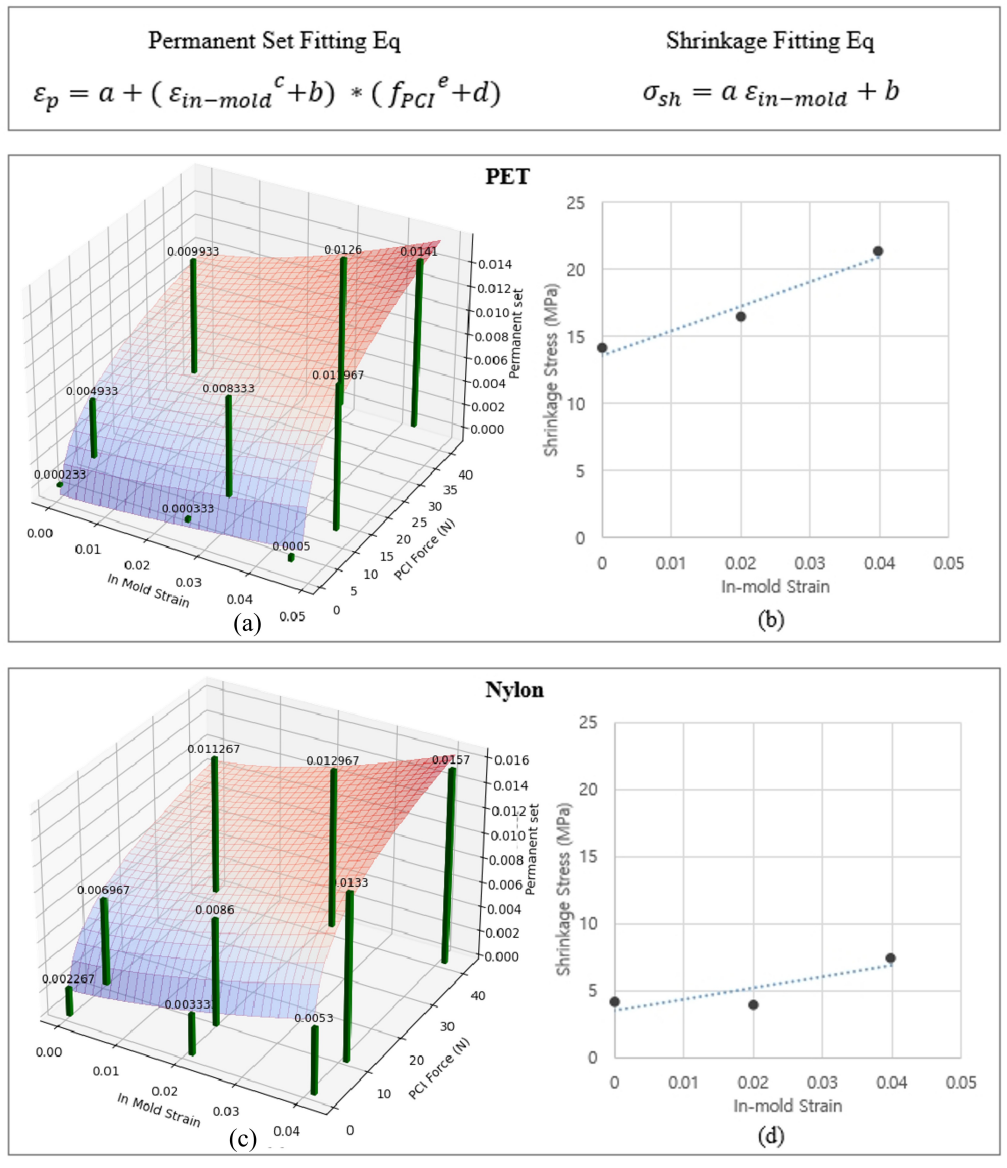
Permanent set surface fitting of PET (**a**) and nylon (**c**) and thermal shrinkage line fitting of PET (**b**) and nylon (**d**) calculated using the fitting equations.

Subsequently, three-dimensional surface fitting, as illustrated in Figure 5, was applied to formulate the relationship between thermal shrinkage, permanent set, and the experimental variables. Three-dimensional graphs visualized how permanent set varied under different loading conditions for both PET cord and nylon cord, materials commonly used in tire construction.

The analysis revealed distinct material-specific behaviors among cord types. PET cord, primarily used in body ply applications, demonstrated significant sensitivity to both PCI force and in-mold strain. Nylon cord, predominantly used in cap ply, exhibited minimal response to variations in in-mold strain while remaining sensitive to PCI force. To accurately represent these material-specific behaviors in FEA, precise mathematical models were developed based on three-dimensional surface fitting using the least-squares method implemented in Python’s Scipy 1.15 library.

This experimental method was applied to various types of cord, including those used in cap ply and body ply applications. Through these systematic experiments, the coefficients for thermal shrinkage and permanent set were derived for each cord type and systematically organized into a comprehensive database. This database served as a critical input for the cured tire shape prediction process in subsequent analyses.

### 2.3. In-Mold Strain Calculation

**Figure 6:**
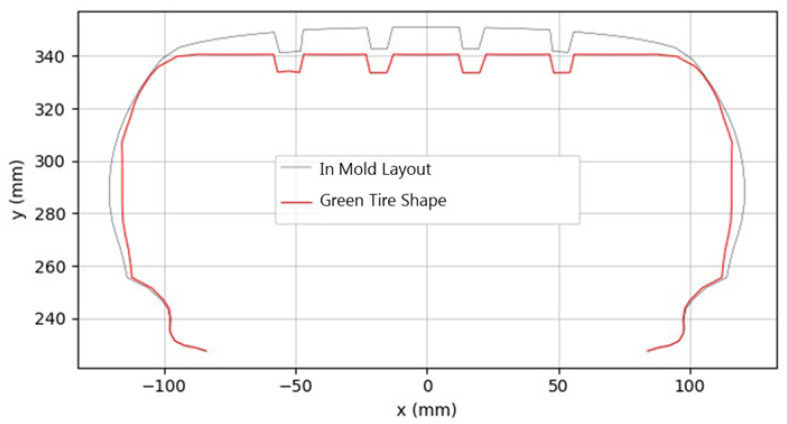
Profile of green tire mesh and in mold layout mesh.

In FEA for tires, the mesh is typically based on the in-mold layout mesh that conforms to the model profile. To accurately simulate the green tire shape, the in-mold layout mesh must be adjusted to match the green tire’s actual dimensions. The green tire shape is characterized by its outer diameter (OD) and section width (SW), which are measured during the manufacturing process. As illustrated in Figure 6, the green tire features distinctive flat tread and sidewall lines.

A critical parameter affecting the green tire OD is the mold lift ratio—a manufacturing parameter that quantifies the vertical displacement applied when removing the cured tire from the building drum. This relationship can be mathematically expressed as follows:

(3) $$GreenTireOD=\frac{Mold\;OD}{Mold\;Lift\;Ratio+1.0}$$

The transformation from the green tire shape mesh to in-mold layout mesh involves applying displacement boundary conditions to each node in the FEA model. During this transformation analysis, the strain experienced by each cord element is calculated and defined as “in-mold strain”. These calculated strain data are then collected for a comprehensive evaluation.

**Figure 7:**
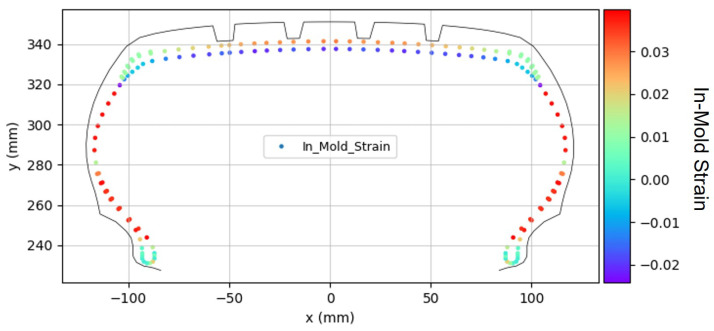
In-mold strain distribution.

The analysis of the in-mold strain distribution revealed significant regional variations across the tire structure. As shown in Figure 7, the greatest deformation occurred in the body ply of the sidewall region. This contrasted with the lower region of the belt, which primarily experienced compressive deformation.

These regional differences in strain distribution are important factors in predicting the final cured tire shape and understanding the mechanical behavior of reinforcement materials during the manufacturing process.

### 2.4. PCI Process Analysis and Thermal Shrinkage

PCI is a process in which air pressure is applied to prevent cord shrinkage and maintain the tire shape after curing in the mold. Since this process begins immediately after the tire is released from the mold, the initial shape for simulating the PCI state starts from the in-mold layout. The PCI analysis process is conducted by applying PCI conditions, including PCI air pressure and PCI rim width, to the in-mold layout shape.

During the PCI process, thermal shrinkage occurs in the fabric cord. As the fabric cord reaches high temperatures during the curing process, it undergoes thermal shrinkage as the temperature decreases in the PCI stage. This thermal shrinkage is calculated based on the in-mold strain occurring in each cord element.

**Figure 8:**
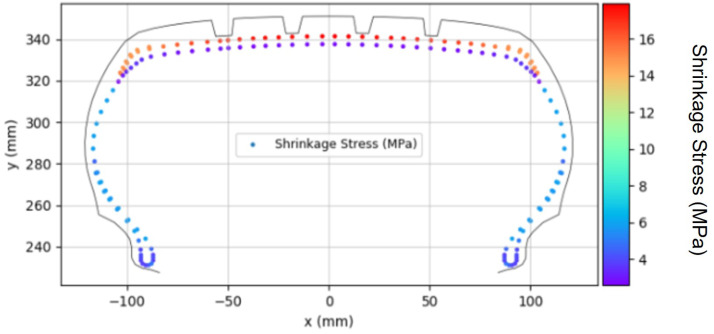
Thermal shrinkage distribution.

To incorporate this thermal shrinkage into the PCI process analysis, it was modeled as compressive stress applied to the fabric cord elements, as depicted in Figure 8. The magnitude of the thermal shrinkage stress was determined using the coefficients obtained from the fabric cord experiments described previously. These coefficients were assigned to each fabric cord element and applied in the simulation to ensure accurate modeling of the thermal shrinkage behavior. This thermal shrinkage behavior was simulated in ABAQUS by applying the experimentally determined shrinkage values as initial stresses to each element.

**Figure 9:**
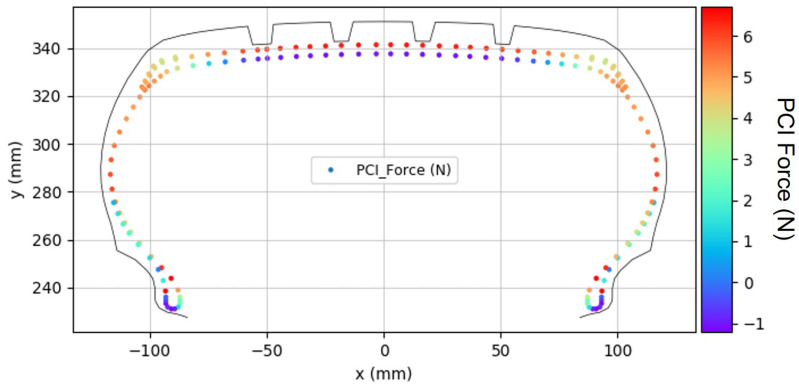
PCI force distribution.

Following the simulation, as shown in Figure 9, the PCI force generated in each cord element was recorded from the analysis results.

The thermal shrinkage influences the growth of OD and SW caused by PCI pressure during the PCI process and ultimately determines the final shape after PCI [7]. Notably, nylon exhibits a relatively greater thermal shrinkage compared to PET. As a result, cap ply, which primarily uses nylon, experiences a higher degree of thermal shrinkage than body ply, which is typically composed of polyester [9,10].

### 2.5. Cured Tire Shape Prediction

The shape of a tire without inflation pressure is affected by gravity, causing sagging and distortion, making it unsuitable as a reference for defining the standardized cured tire shape. Therefore, the cured tire shape is generally defined at 10% inflation pressure.

**Figure 10:**
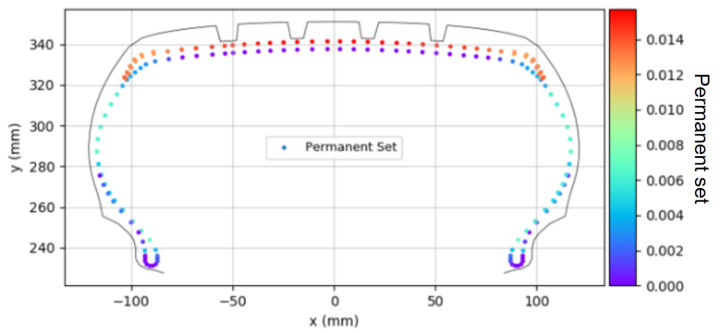
Permanent set distribution.

Figure 10 displays the distribution of the initial permanent set occurring at 10% inflation pressure. The distribution of the permanent set in each fabric cord was calculated using the fitting equation derived in Figure 5, based on the history of in-mold strain and PCI force at each section. This distribution of the permanent set ultimately determined the cured tire shape. This permanent set behavior was simulated in ABAQUS by applying the experimentally determined permanent set values to each element using the “Plastic” material model keyword.

Additionally, the final profile at 100% inflation pressure must match the actual tire shape to be considered an accurate cured tire shape. Hence, this study primarily focused on the tire profile at 100% inflation pressure while also comparing it with the profile at 10% inflation pressure.

**Figure 11:**
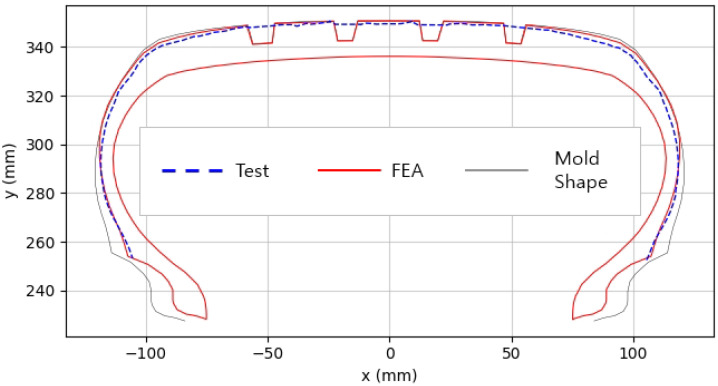
Comparison of 10% inflation pressure profiles between test and FEA result.

**Figure 12:**
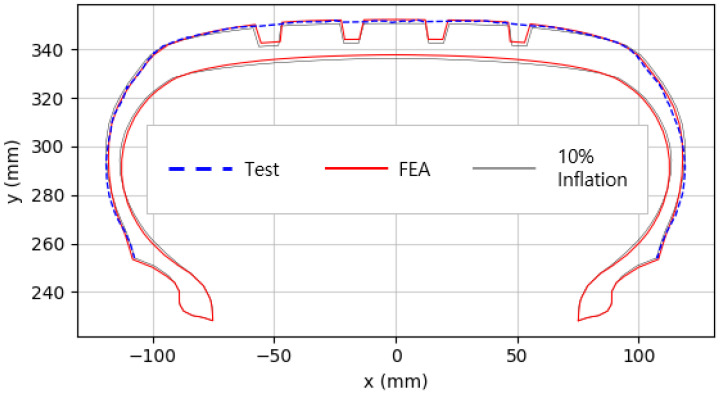
Comparison of 100% inflation pressure profiles between test and FEA result.

The shape obtained from the analysis at 10% inflation pressure was defined as the cured tire shape. From this cured tire shape, inflation pressure was gradually increased to 100% to obtain the final tire shape at full inflation. Figure 11 and Figure 12 present comparison graphs of the profiles between the experimental results and the FEA results for both 10% and 100% inflation pressure conditions, respectively.

As shown in Figure 11, the actual tire profile at 10% inflation pressure, represented by the blue dotted line, and the FEA result, represented by the red solid line, exhibited a slight discrepancy in the shoulder area, while the rest of the profile showed good agreement. In Figure 12, the actual tire profile at 100% inflation pressure and the FEA result closely matched with minimal deviation across all regions, confirming the accuracy of the simulation in predicting the final tire shape.

## 3. Results and Discussion

This study analyzed a total of 65 tire profile test results to evaluate the effectiveness of the cured tire shape prediction method against conventional approaches. The comparison examined three key dimensions: OD, SW, and overall profile. Table 2 summarizes the errors between experimental and simulation results for OD and SW.

The implementation of the cured tire shape prediction method yielded substantial improvements in dimensional accuracy. Specifically, the OD error decreased from 3.68 mm to 1.69 mm, representing a reduction of 1.99 mm (54.2%). Similarly, the SW error decreased from 3.11 mm to 1.57 mm, showing a reduction of 1.54 mm (49.5%).

The maximum error for OD was less pronounced compared to SW when applying the cured tire shape prediction method. This difference stems from the fact that OD is primarily influenced by the steel belt rather than the fabric cord in the body ply. Conversely, SW dimensions are largely determined by the fabric cord in the body ply, explaining the more substantial error reduction observed.

Table 3 presents a comparison of FEA results for six sample tires at 100% inflation pressure. The conventional FE-predicted profiles (without the use of the cured tire shape prediction method) showed notable deviations from actual profile test results, represented by blue dotted lines. Meanwhile, profiles generated using the cured tire shape prediction method demonstrated remarkably better alignment with test results, highlighting the method’s superior accuracy.

The profile shape directly influences footprint geometry. The enhanced prediction method developed in this study significantly improved footprint FEA accuracy, as shown in Table 4, where the predicted footprints closely corresponded to experimental measurements. Footprint analyses using the cured tire shape prediction method exhibited marked improvements, especially regarding shoulder-side footprint block length, which more faithfully reproduced the square-shaped patterns observed in testing.

Traditional FEA methods without the prediction algorithm tend to generate elongated center block lengths, resulting in more rounded footprint shapes that deviate from actual test data.

Test results for samples No. 1, 5, and 6 displayed distinctly square-shaped footprints. Traditional FEA simulations produced shorter shoulder-side footprint blocks, creating overly rounded shapes. The advanced prediction method, however, generated appropriately elongated shoulder-side footprint blocks, accurately reproducing the square-shaped footprints observed during testing.

For samples No. 2, 3, and 4, where test footprints exhibited naturally rounded shapes, both simulation approaches yielded generally rounded profiles. Nevertheless, the cured tire shape prediction method reproduced detailed block patterns with substantially higher fidelity compared to conventional methods, demonstrating its versatility across various footprint geometries.

Despite the significant improvements demonstrated, the current methodology has several limitations that warrant acknowledgment. First, the thermal shrinkage of rubber compounds was not directly incorporated into the model. Instead, the deformed shape due to thermal effects was estimated based on statistical data of rubber shrinkage and expansion patterns in tires, rather than through direct physical modeling of these complex phenomena. This simplification, while practical for implementation, may introduce discrepancies in scenarios where rubber compound behavior dominates the deformation process.

Second, computational constraints affected material modeling choices in ABAQUS. The use of a static, 2D axisymmetric analysis was necessary for ensuring numerical convergence within a short time for the practical implementation, but this approach cannot fully capture the tread pattern effects present in fully patterned 3D tire models. Consequently, the methodology may show reduced accuracy when applied to tires with complex tread designs where local deformations significantly influence overall shape.

Third, the experimental characterization focused primarily on standard operating conditions. The model may exhibit reduced accuracy when applied to extreme temperature conditions or unconventional loading scenarios that fall outside the experimental parameter space used for model development. Furthermore, the method’s effectiveness for specialty tire types with unusual cord arrangements or innovative materials remains untested.

## 4. Conclusions

This study experimentally established that permanent set and thermal shrinkage of fabric cord during tire manufacturing were primarily determined by in-mold strain and PCI force. Based on these findings, a comprehensive cured tire shape prediction method was developed and validated through finite element analysis. The key contributions of this research can be summarized as follows:

1. Material property relationships: the permanent set and thermal shrinkage of fabric cord in tire manufacturing are functions of in-mold strain and PCI force, with material-specific response patterns identified for different cord types.
2. Shape determination mechanism: the final shape of cured tires is significantly influenced by the distribution of permanent set and thermal shrinkage across fabric cord components.
3. Experimental integration: through systematic experimentation, quantitative values for permanent set and thermal shrinkage were determined and successfully incorporated into a finite element analysis, enabling more accurate tire shape prediction.
4. Enhanced predictive accuracy: FEA incorporating the cured tire shape prediction method demonstrated substantial improvements in accuracy compared to conventional approaches:
  - A 54.2% reduction in outer diameter (OD) error;
  - A 49.5% reduction in section width (SW) error;
  - Significantly improved profile prediction and footprint pattern fidelity.

The methodology proposed in this study represents a significant advancement in tire shape prediction accuracy with demonstrated consistency against experimental results. These improvements have important implications for tire design and manufacturing processes. The enhanced predictive capabilities enable more precise tire design optimization, while improved footprint prediction allows for a better assessment of tire–road contact characteristics. More accurate dimensional predictions can reduce prototype iterations and associated development costs, leading to more efficient product development cycles and potential material savings.

To build upon the foundation established in this study, future research should apply the cured tire shape prediction method to a broader range of tire designs and operating conditions. Investigating the impact of additional manufacturing parameters on tire shape prediction would further refine the methodology. Extending the approach to dynamic performance predictions, including rolling resistance, traction, and wear characteristics, would connect shape prediction to functional performance metrics. Developing integrated computational workflows that combine the cured tire shape prediction with other performance simulation methods would create a more comprehensive design toolset. Finally, exploring the application of this approach to emerging tire technologies and alternative materials would ensure the methodology remains relevant as the industry evolves. This research represents a significant step toward bridging the gap between tire design and manufacturing considerations, providing valuable tools for enhancing both tire performance and production efficiency.

## Figures and Tables

**Table 1:** Experiment conditions with varied in-mold strain and PCI forces. Three specimens were tested under each condition.

Conditions		In-Mold Strain		
		0%	2%	4%
PCI Force	1N	0%, 1N	2%, 1N	4%, 1N
	10N	0%, 10N	2%, 10N	4%, 10N
	40N	0%, 40N	2%, 40N	4%, 40N

**Table 2:** Comparison of OD and SW errors between test and FEA results.

Average Error	Not Use	Use	STD. Error	Not Use	Use
OD (mm)	3.68	1.69	OD (mm)	2.43	1.17
SW (mm)	3.11	1.57	SW (mm)	2.22	1.35

**Table 3:** Comparison of 10% and 100% inflation pressure profiles between test and FEA results.

No.	Not Using Cured Tire Shape Prediction	Using Cured Tire Shape Prediction
1	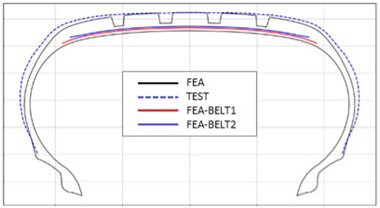	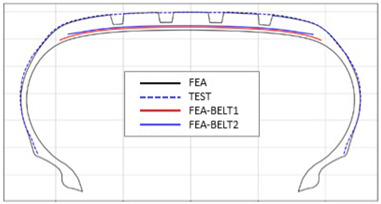
	Size: 235/55R18, Inflation Pressure: 1.8 kgf/cm^2^	
2	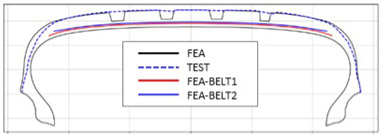	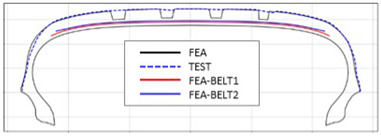
	Size: 265/35R20, Inflation Pressure: 2.3 kgf/cm^2^	
3	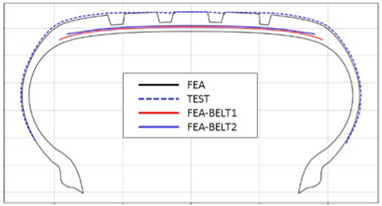	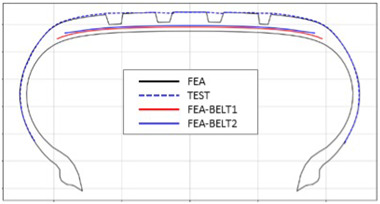
	Size: 235/55R19, Inflation Pressure: 2.5 kgf/cm^2^	
4	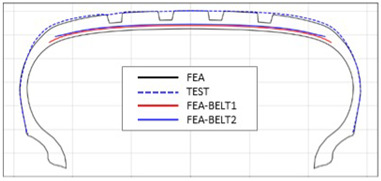	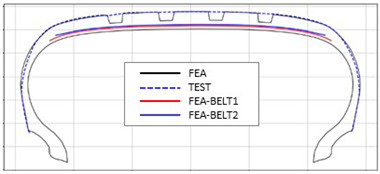
	Size: 285/45R21, Inflation Pressure: 2.3 kgf/cm^2^	
5	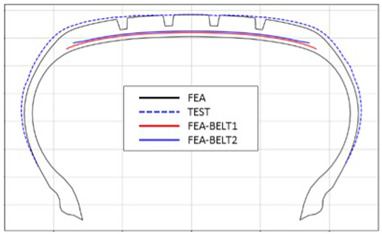	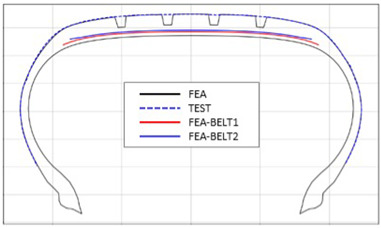
	Size: 235/60R18, Inflation Pressure: 2.5 kgf/cm^2^	
6	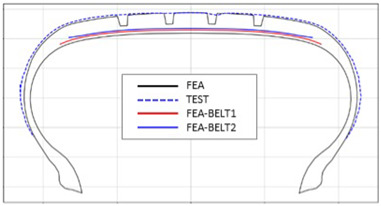	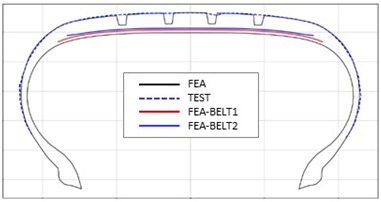
	Size: 215/55R17, Inflation Pressure: 1.8 kgf/cm^2^	

**Table 4:** Comparison of footprint between test and FEA results.

No.	FEA Result (Without Cured Tire Shape Prediction)	FEA Result (With Cured Tire Shape Prediction)	Actual Test Result
1	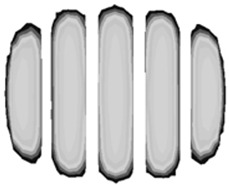	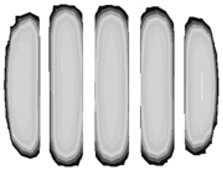	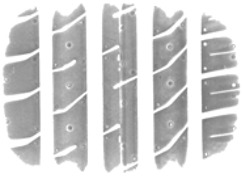
	Size: 235/55R18, Inflation Pressure: 1.8 kgf/cm^2^		
2	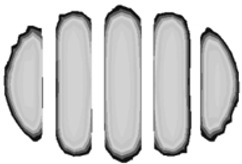	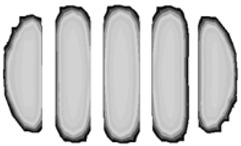	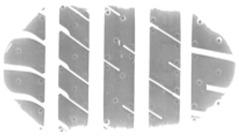
	Size: 265/35R20, Inflation Pressure: 2.3 kgf/cm^2^		
3	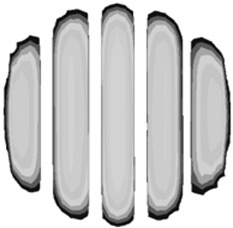	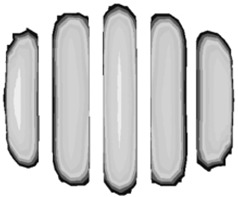	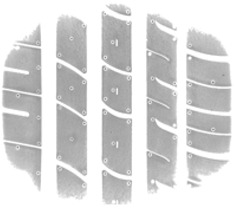
	Size: 235/55R19, Inflation Pressure: 2.5 kgf/cm^2^		
4	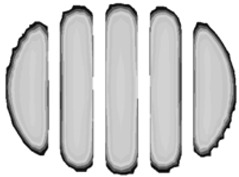	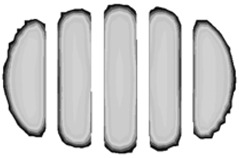	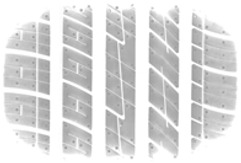
	Size: 285/45R21, Inflation Pressure: 2.3 kgf/cm^2^		
5	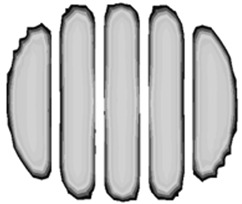	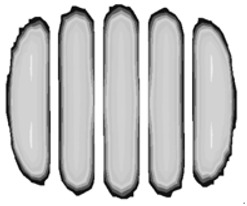	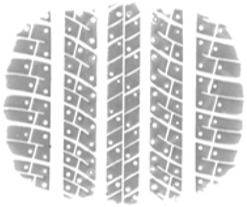
	Size: 235/60R18, Inflation Pressure: 2.5 kgf/cm^2^		
6	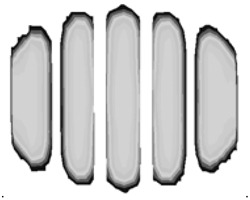	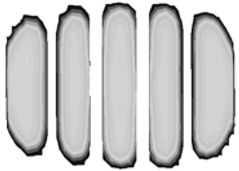	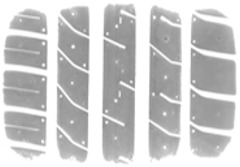
	Size: 215/55R17, Inflation Pressure: 1.8 kgf/cm^2^		

## Data Availability

Data are contained within the article.

## Abbreviations

The following abbreviations are used in this manuscript:

FEA	Finite element analysis
PCI	Post-Cure Inflation
OD	Outer diameter
SW	Section width
